# Double Ventricular Responses Leading to Reversible Cardiomyopathy as Late Complication after Slow-Pathway Ablation

**DOI:** 10.1155/2015/326576

**Published:** 2015-09-30

**Authors:** Umut Celikyurt, Meinrad Gawaz, Juergen Schreieck, Peter Seizer

**Affiliations:** Medizinische Klinik III, Kardiologie und Kreislauferkrankungen, Eberhard Karls Universität, Tübingen, Germany

## Abstract

Double ventricular response is a rare arrhythmia that results from simultaneous antegrade conduction over the fast and slow pathways of AV node. Double ventricular responses may lead to arrhythmia-related cardiomyopathy. As far as we know, there is not any reported reversible cardiomyopathy development that resulted from double ventricular responses to one atrial impulse after slow pathway ablation. We report a unique case of double ventricular response cardiomyopathy that has been developed 5 years after slow pathway ablation.

## 1. Introduction

Double ventricular response is a rare arrhythmia that results from simultaneous antegrade conduction over the fast and slow pathways of AV node. Slow-pathway conduction must be slow enough to allow his-purkinje system to recover excitability after depolarization by the first excitation over the fast pathway. Double ventricular responses may lead to tachycardia-related cardiomyopathy. We report a unique case of double ventricular response cardiomyopathy that has been developed 5 years after slow-pathway ablation.

## 2. Case Report

**Figure 1:**
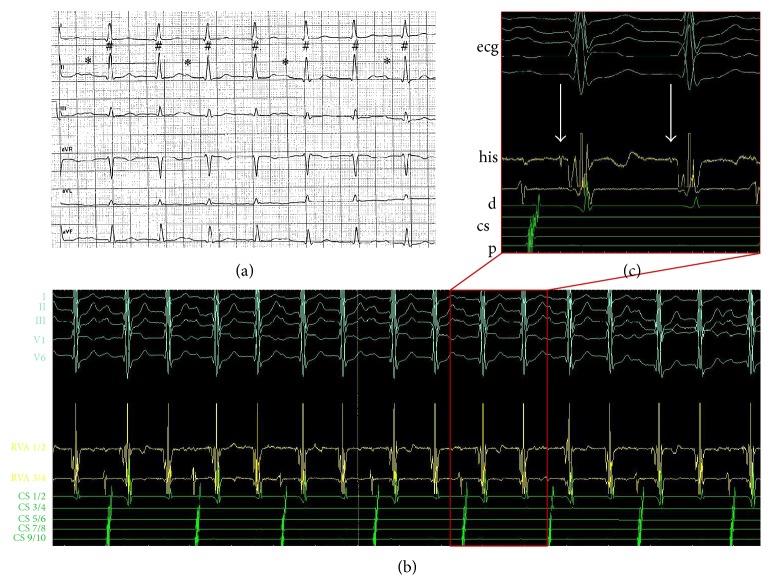
(a) ECG showing two ventricular beats (#) after one atrial beat (*∗*); (b) intracardiac electrocardiogram showing two ventricular beats after one atrial beat; (c) intracardiac electrocardiogram revealed his potential before each ventricular signal (see arrows). ECG: electrocardiography.

**Figure 2:**
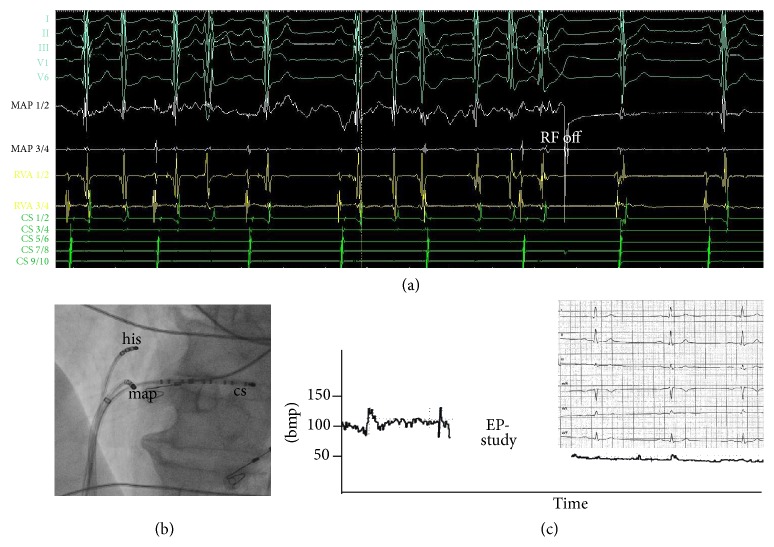
(a) Intracardiac electrocardiogram showing disappearance of the arrhythmia during ablation therapy with radiofrequency (RF). (b) Fluoroscopy (45° LAO) showing ablation catheter in slow-pathway position during ablation. (c) Drop in heart rate after successful ablation in continuous heart rate monitoring and postablation ECG showing normal sinus rhythm. ECG: electrocardiography, CS: coronary sinus, bmp: beats per minute, and RF: radiofrequency.

A 69-year-old patient admitted to our ambulance with severe dyspnea. Five years ago a slow-pathway cryoablation due to typical atrioventricular nodal reentry tachycardia was performed. Transthoracic echocardiography performed at admission revealed severely reduced left ventricular ejection fraction (LVEF) (30%), which was reported normal 5 years ago. In the next step, haemodynamic relevant stenoses were excluded in coronary angiography. Cardiac MRI confirmed severely reduced LVEF without the presence of myocardial fibrosis. Electrocardiography (ECG) revealed an uncommon finding of two ventricular beats after one atrial beat (Figure 1(a)). An electrophysiological study was performed due to this uncommon ECG. ECG revealed a double ventricular excitation due to a dual AV-node conduction capacity (Figures 1(b) and 1(c)). Slow-pathway ablation was performed again with radiofrequency using a solid tip catheter (4 mm, 30 watt, Figures 2(a) and 2(b)). Junctional beats appeared 15 seconds after start of ablation, the second ventricular excitation terminated, and a stable sinus rhythm was established. The heart rate dropped from 100 bpm (double ventricular response) to about 50 bpm in telemetric monitoring and ECG revealed normal sinus rhythm (Figure 2(c)). Interestingly, the LVEF was improved to 40% after two months. After 12 months LVEF was completely normalized and left ventricular end-diastolic diameter was decreased from 59 mm to 53 mm.

To the best of our knowledge, this is the first report of reversible cardiomyopathy due to double ventricular response to one atrial impulse that developed as a late complication of slow-pathway cryoablation.

## 3. Discussion

Atrioventricular nodal reentrant tachycardia (AVNRT) is one of the most common sustained supraventricular arrhythmia. Catheter ablation of the slow pathway is treatment of choice for patients with recurrent drug-refractory AVNRT [1]. The risk of periprocedural atrioventricular block is a known complication and less than 1% of patients treated with radiofrequency ablation have been reported [2]. As far as we know, there is not any reported reversible cardiomyopathy development that resulted from double ventricular responses to one atrial impulse after slow-pathway ablation.

Double ventricular response is a rare arrhythmia that results from simultaneous antegrade conduction over the fast and slow pathways of AV node [3]. Slow-pathway conduction must be slow enough to allow his-purkinje system to recover excitability after depolarization by the first excitation over the fast pathway. Also, there is unidirectional retrograde block in fast and slow pathways [4].

The presence of his electrograms before each QRS complex and a relatively stable interval between the fast- and slow-pathway his electrograms during electrophysiological study supports the concept. However, simple junctional extrabeats as bigeminus cannot fully be excluded. The termination of the tachycardia with slow-pathway ablation is the treatment of choice.

Although double ventricular responses and tachycardia-related cardiomyopathy that reverses with slow-pathway ablation has been described in the literature, our case is unique in which double ventricular responses and related cardiomyopathy occurred 5 years after slow-pathway ablation.

It is known that there may be late recovery of conduction after successful ablation [5]. In our patient, the delayed development of dual ventricular responses to one atrial stimulus after slow-pathway ablation may result from partial restoration of electrophysiological function at slow pathway.

The normalization of LV function after ablation strongly supports that cardiomyopathy was related to the dual ventricular responses.
